# Mapping the Hot Spots and Evolution Main Path of Whole-Body Vibration Training Since the 21st Century: A Bibliometric Analysis

**DOI:** 10.3389/fbioe.2022.920846

**Published:** 2022-07-11

**Authors:** Dan Dong, Mingli Sun, Dan Xu, Shuang Han, Liyuan Cui, Shu Cao, Ying Yang, Shuang Xu

**Affiliations:** ^1^ Department of Pathophysiology, College of Basic Medical Science, China Medical University, Shenyang, China; ^2^ School of Kinesiology, Shenyang Sport University, Shenyang, China; ^3^ Library of China Medical University, Shenyang, China

**Keywords:** whole-body vibration training (WBVT), bibliometric analysis, visualization, citation chronology map, main path analysis, hot spots, biclustering analysis

## Abstract

To evaluate the global scientific output of the research on whole-body vibration training (WBVT) and explore the current status and trends in this field over the past decades using bibliometric methods, we retrieved the literature related to WBVT from 2000 to the present in the Web of Science Core Collection (WoSCC). We analyzed annual publications, citations, countries, organizations, productive authors, and source 14 journals by the Web of Science online bibliometric analysis. We visualized the WBVT research trends and explored influential organizations and authors through VOSviewer. Then, we constructed a citation chronology map by HistCite to obtain the knowledge base of this field and made a primary citation path analysis by Pajek. Finally, we mined the hot spots of WBVT by BICOMB and gCLUTO. Overall, there were 1,629 publications included in this study between 2000 and 2022. The United States contributed the most publications in this field, and the country with the most active partnership was Spain. The University of Cologne ranked highest among top productive organizations. Bernardo-Filho, Mario, from Universidade do Estado do Rio de Janeiro, ranked first among the top productive authors. The Journal of Strength and Conditioning Research topped the list of journals with the most publications on WBVT by a wide margin. The WBVT research field started from Rittweger’s study on the acute physiological effects of WBVT in 2000, which was divided into two stages. The first stage focused on improving athletic ability, and the second stage gradually turned to the application in medicine. A keyword analysis showed the exercise rehabilitation of several aging chronic diseases was the research trend and hot spot of WBVT. The current study provided a time-based development and a global network hub for WBVT research, contributing to identifying core target diseases of WBVT and providing various insights for researchers in the future.

## Introduction

Whole-body vibration training (WBVT), a non-invasive physical therapy, offers a passive exercise stimulation by vibration platforms and has become a reasonable procedure for enhancing muscular performance (Godinez et al., 2014; Annino et al., 2017). WBVT induces adaptive neuromuscular changes by producing biomechanical and physiological effects. Several factors determine its impact, including vibration mode and parameters and training posture and training frequency (Arias et al., 2009; Tankisheva et al., 2014).

WBVT is a standard training method for improving athletic performance and maintaining astronauts’ skeletal muscle mass and strength, which gradually becomes a focus in exercise rehabilitation (Song et al., 2019). Previous research has investigated the effects and mechanisms of WBVT for various medical conditions. Clinical studies indicated that WBVT could be applied to the rehabilitation of several chronic diseases including stroke, diabetes, osteoarthritis, and musculoskeletal pain (Wang et al., 2020; Rodríguez-Reyes et al., 2021; Wei and Cai, 2021; Qiu et al., 2022). Furthermore, fundamental research has investigated the underlying mechanisms of WBVT on muscle strength and performance, bone mineral content, and bone mineral density (Bleeker et al., 2005; Machado et al., 2010). Considering the increasing number and the diversity of published studies, an urgent bibliometric analysis on WBVT is needed to explore the hotspots and predict the future trends of WBVT research.

Bibliometric analysis is a useful tool for the quantitative and qualitative analysis of published academic literature for tracking hotspots and predicting future trends in a specific research field. It uses mathematical and statistical tools to enable the identification of influential publications, journals, countries, organizations, and researchers in a specific research area and assess their relationships and impacts (Van Eck and Waltman, 2010; Jain et al., 2015). It provides data to inform policymaking and clinical guidelines in the future (Zhao et al., 2018). Bibliometric analyses have been used to track hotspots and predict research trends in various fields, including exercise and rehabilitation (Park et al., 2020; Park et al., 2021; Xiang et al., 2021). With the rapid development in exercise rehabilitation techniques, growing attention has been concentrated on WBVT, and more and more articles on this topic have been published in recent years. However, there has not been any bibliometric analysis on WBVT until now. In the present study, we searched the studies on WBVT published during 2000–2022 and conducted a bibliometric analysis to dig the hotspots and predict the trends in this field.

## Materials and Methods

### Data Source and Acquisition Process

Given the consideration of the quality of the literature, as well as the requirement for reference format in 64 citation analysis (Gao et al., 2021; Wu et al., 2021a; Wu et al., 2021b), we adopted the Web of Science 65 Core Collection (WoSCC) (editions: Science Citation Index Expanded [SCIE], and Social Science Citation 66 Index [SSCI]) as the data source. Given the different English expressions of “whole-body vibration training,” this study referred to the keywords that were selected by relevant researchers to extract the literature related to WBVT effectively and tried to make the search results accurately include the research literature on WBVT in WoSCC. After multiple searches and comparisons, we finally determined the search formula as “TS = Whole-body Vibration Train* OR Whole body Vibration Train* OR Whole-body Vibration Exercise* OR Whole body Vibration Exercise* OR Whole-body Vibration Therap* OR Whole body Vibration Therap*) OR TS = (WBV NEAR Train*) OR TS = (WBV NEAR Exercise*) OR TS = (WBV NEAR Therap*) OR TS = (WBVT OR WBVE).”

On 7 May, 2022, the search command was carried out in the “advanced search” of WoSCC (SCIE and SSCI). In order to obtain the literature with a complete structure which was suitable for analysis tools, we selected document types as “Articles” and “Review Articles” in “Refine results” (Zhai et al., 2021). There were no restrictions on the languages, countries/regions, affiliations, journals, research areas, and Web of Science categories. A total of 1,883 initial publications since the 21st century were retrieved. The search results were independently screened by two researchers (MS and DD) in the related discipline, and all the discrepancies were resolved by the principal investigator (SX). We removed literature not related to medicine, sports, exercise, physical science, or rehabilitation, and literature mainly studying local vibration. We exported the final 1,629 literature records to a plain text file with “Full Record and Cited References.”

### Analysis Tools and Research Methods

Bibliometric analysis, citation analysis, and content analysis were used to analyze the obtained literature. Bibliometrics mainly uses statistical and mathematical methods to study the external formal features of the literature to master the quantitative relationship, distribution structure, and development trend (Thompson and Walker, 2015). Citation analysis is an informetric method to analyze the citation and cited phenomena of scientific journals, publications, authors, and other analysis objects to reveal their quantitative characteristics and internal laws (Boyack and Klavans, 2010). Content analysis studies the content features of the literature, which is to analyze and ratiocinate the relevant literature in a certain period and obtain the research hot spots through clustering (Ormerod, 2010).

VOSviewer (version 1.6.17) was used to visualize bibliometrics in this study. VOSviewer (https://www.VOSviewer.com/) is a knowledge mapping tool developed by Van Eck and Waltman from the Centre for Science and Technology Studies (CWTS) of Leiden University (Van Eck and Waltman, 2010). In the knowledge maps generated by VOSviewer, an element is formed by a circle and a label. Many factors determine the size of an element, including the degree of nodes, the strength of the connections, the number of references, etc. Links between the nodes can represent the relationship between elements. VOSviewer draws a feature map by analyzing the external features of the literature. The topic co-occurrence map can find the structural distribution of research hot spots. The authors’ cooperation map can find research groups. The authors’ coupling network can find the similarities and differences of scholars on research topics, etc. (Qiu et al., 2014). We chose “create a map based on bibliographic data” to construct the co-authorship maps of countries, organizations, and authors, the co-citation map of journals, and the co-occurrence map of keywords. The counting method used in this study was “Full Counting.” Two standard weight attributes of “Link” and “Total link strength” were applied.

The citation analysis shows the relationship between different literature and maps and describes the history of WBVT. This study used HistCite to locate important literature and academic experts in this field. Using HistCite developed by Garfield, inventor of SCI, we analyzed and reorganized the retrieval results of WoSCC and displayed the chronological sequence and cross-reference relationship of critical events on the chronological map. The chronological citation map enabled us to quickly understand the historical development and change trend of WBVT. In this study, HistCite Pro (version 2.1) was used for context sorting and visual analysis of the literature about WBVT since the 21st century. HistCite Pro (version 2.1) (https://zhuanlan.zhihu.com/p/20902898/) developed by Zhaofeng Luo from the University of Science and Technology of China, was used to conduct an analysis based on LCS (Local Citation Score), including TGCS (Total Global Citation Score) and TLCS (Total Local Citation Score) (https://zhuanlan.zhihu.com/p/20902898/). We constructed the historiography for WBVT based on both TGCS and TLCS and identified the key literature (Arunachalam and Viswanathan, 2008). We used the following function in the Graph Maker Menu of HistCite Pro to select records to include in a graph. “LCS count limit: 30” would select the top 30 records from the collection sorted by LCS. This is usually the best way to show the most crucial citation links within the collection. Graph data were saved to a “.net” file, and the format “Pajek 1” containing detailed node information was suitable for analysis and further manipulation with Pajek. The citation primary path analysis can identify the most relevant literature in the whole citation flow and emphasize which publications are the development core of a particular topic and have a significant impact in the development process of the topic (Olczyk, 2016). Critical literature is an essential part of reconstructing research in the scientific field. Pajek (http://vlado.fmf.uni-lj.si/pub/networks/pajek/) developed by Vladamir Batagelj from the University of Ljubljana, is a network analysis and visualization program specifically designed to work with large datasets and generate a series of crossover networks to analyze and examine the evolution of the network (Batagelj and Mrvar, 2004). This study used Pajek (version 5.14) software for the citation main path analysis. In the “Networks” window of Pajek (version 5.14), we loaded the ". net” file obtained from HistCite Pro, and created an “Acyclic Network” with “Critical Path Method-CPM” to get the main path of WBVT.

A content analysis was used to mine the hot spots of WBVT. The content of a publication is the topic that it deals with, which is generally expressed by the abstract, classification number, and keywords. We introduced the co-word biclustering analysis to explore the relationship between the literature and the high-frequency words. Data extraction and matrix construction were implemented by BICOMB (version 2.1) (Bibliographic Items Co-occurrence Matrix Builder), developed by Lei Cui from China Medical University (Lei et al., 2008). BICOMB (https://www.cmu.edu.cn/dmi/nr.jsp?urltype = news. NewsContentUrl&wbtreeid = 2161&wbnewsid = 4589) may accurately extract, classify, store, and count the items of bibliographic information, and generate a co-occurrence matrix to provide comprehensive, accurate, and authoritative primary data for further research. A term-paper matrix is a binary matrix that uses 0 and 1 to indicate whether the term appears in the article. Biclustering analysis can realize the clustering of rows and columns simultaneously, which is performed using gCLUTO (version 1.0), developed by Matt Rasmussen and Mark Newman from Minnesota University (Rasmussen and Karypis, 2003). GCLUTO (http://glaros.dtc.umn.edu/gkhome/cluto/gcluto/download/) is specially used for visual analysis of the co-word matrix. Research hot spots are expressed as matrix and mountain visualization through the clustering analysis. A price equation was used to determine the threshold of the high-frequency keywords (Liu et al., 2017).

$$\text{M}=0.749\sqrt{\text{Nmax}},$$

where Nmax represents the highest frequency of the keyword in the dataset. M represents the threshold of the high-frequency keywords. In this study, Nmax = 474, so $\text{M}=0.749\sqrt{474}\approx 16.3$ . We imported a high-frequency keyword-paper matrix with a frequency greater than or equal to 16 into gCLUTO. We chose “Repeated Bisection” for “Cluster Method,” “I2” for “Criterion Function,” “Cosine” for “Similarity Function,” and “10” for “Number of Iterations.” The clustering results were used to create “Matrix Visualization” and “Mountain Visualization.” In the tree visualization, the left side of the row clustering was dendritical in structure of the keywords, and the right side was the corresponding keywords. Column clustering was the clustering of literature co-occurrence with the high-frequency keywords. The color of the square in the visualization was used to describe the value of the original matrix data. The deeper the red, the higher the frequency. The three-dimensional mountain visualization was used to describe the overall characteristics of each cluster. A mountain represented a cluster. The height of the mountain described the similarity within the cluster. Mountain volume described the amount of literature in the cluster. Only the color of the peak was meaningful, which was used to describe the standard deviation within the cluster. Deeper red represented a lower standard deviation, while blue was the opposite.

## Results

### Overall and Annual Distribution of Publications

**FIGURE 1:**
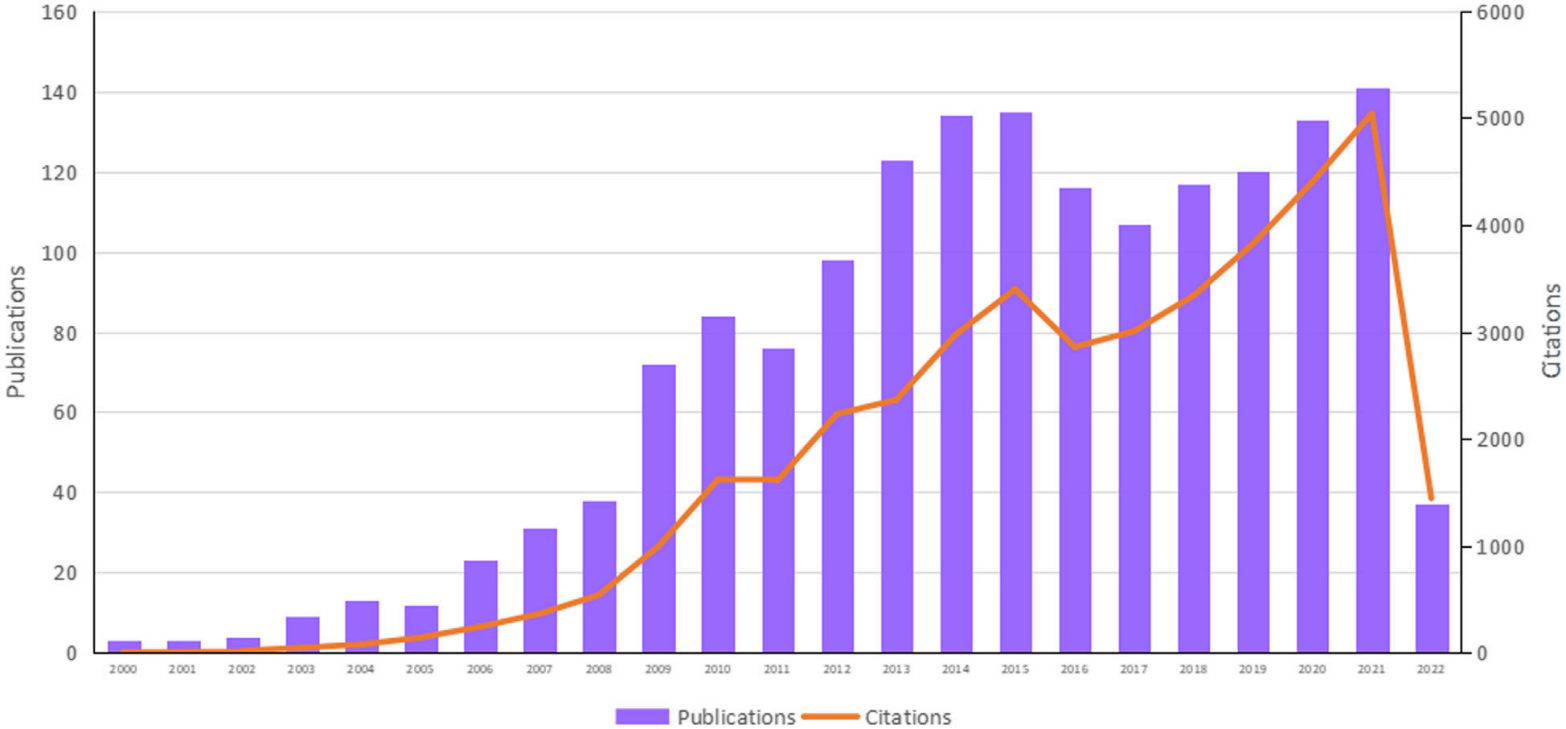
Times cited and publications over time in WBVT research since the 21st century. (Citations refer to the times cited in the year when the publication was issued).

A total of 1,883 original literature files on the topic of WBVT consisted of 1,575 articles, and 308 reviews were retrieved in the database of WoSCC, including 5 ESI (Essential Science Indicators) highly cited articles (Table 1). The document types excluded were meeting abstracts, letters, editorial materials, corrections, retractions, and news items. Among the 1,883 original literature files, 254 were excluded for the following reasons: 1) the research topics were not related to medicine, sport, exercise, physical science, or rehabilitation, and 2) local vibrations dominated the research topics. 1,629 literature files were eventually included. Since the 21st century, the research of WBVT has been developed gradually. The amount of literature and the frequency of citations were three and two in 2010. It increased to 141 and 5,044 in 2021, indicating that the number of publications and the frequency of citations both increased (Figure 1).

**TABLE 1:** ESI highly cited articles in the field of WBVT.

Rank	Frequency	Article title	Journal	IF	Document type	Representative author/publication year
1	556	Exercise for preventing and treating osteoporosis in postmenopausal women.	Cochrane Database of Systematic Reviews	5.912	Review	Howe, TE/2011
2	526	Interventions for preventing falls in older people living in the community.	Cochrane Database of Systematic Reviews	5.785	Review	Gillespie, LD/2012
3	301	Sarcopenic obesity in older adults: aetiology, epidemiology, and treatment strategies.	Nature Reviews Endocrinology	24.646	Review	Batsis, JA/2018
4	288	Exercise for improving balance in older people.	Cochrane Database of Systematic Reviews	5.912	Review	Howe, TE/2011
5	172	The Importance of muscular strength: training considerations.	Sports Medicine	7.583	Review	Suchomel, TJ/2018

### Distribution of Regions and Co-Authorship of Countries

Based on the web of science online bibliometric analysis, the 1,629 publications on WBVT originated from 61 different countries. The top 10 countries participating in the WBVT study have 1,475 records, accounting for 91% of the total publications (Table 2). Due to cooperation among countries, the top 10 countries may publish less than 1,475 articles. The United States ranked No. 1 which contributed the most publications (*n* = 303, 18.6%), followed by Germany (*n* = 218, 13.4%), China (*n* = 191, 11.7%), and Spain (*n* = 188, 11.5%). The United States had the highest number of citations with 8,802, followed by Britain (6,549 citations), and Germany (5,640 citations). It can be seen from Table 2 that the frequency of citations is not completely proportional to the amount of literature unless a country has an absolute advantage in the literature output, such as the United States. Belgium (3,814 citations) ranked fourth in citations higher than Spain (3,746 citations), and it was not in the top 10 for literature output.

**TABLE 2:** Top 10 countries that published the most publications on WBVT.

Rank	Country	Publication count	% of 1,629	Times cited
1	USA	303	18.600	8,802
2	Germany	218	13.382	5,640
3	People’s R China	191	11.725	2,328
4	Spain	188	11.541	3,746
5	Britain	146	8.963	6,549
6	Brazil	144	8.840	1,177
7	Italy	124	7.612	3,360
8	Australia	83	5.095	2,366
9	Canada	78	4.788	2,510
10	France	71	4.359	881

**FIGURE 2:**
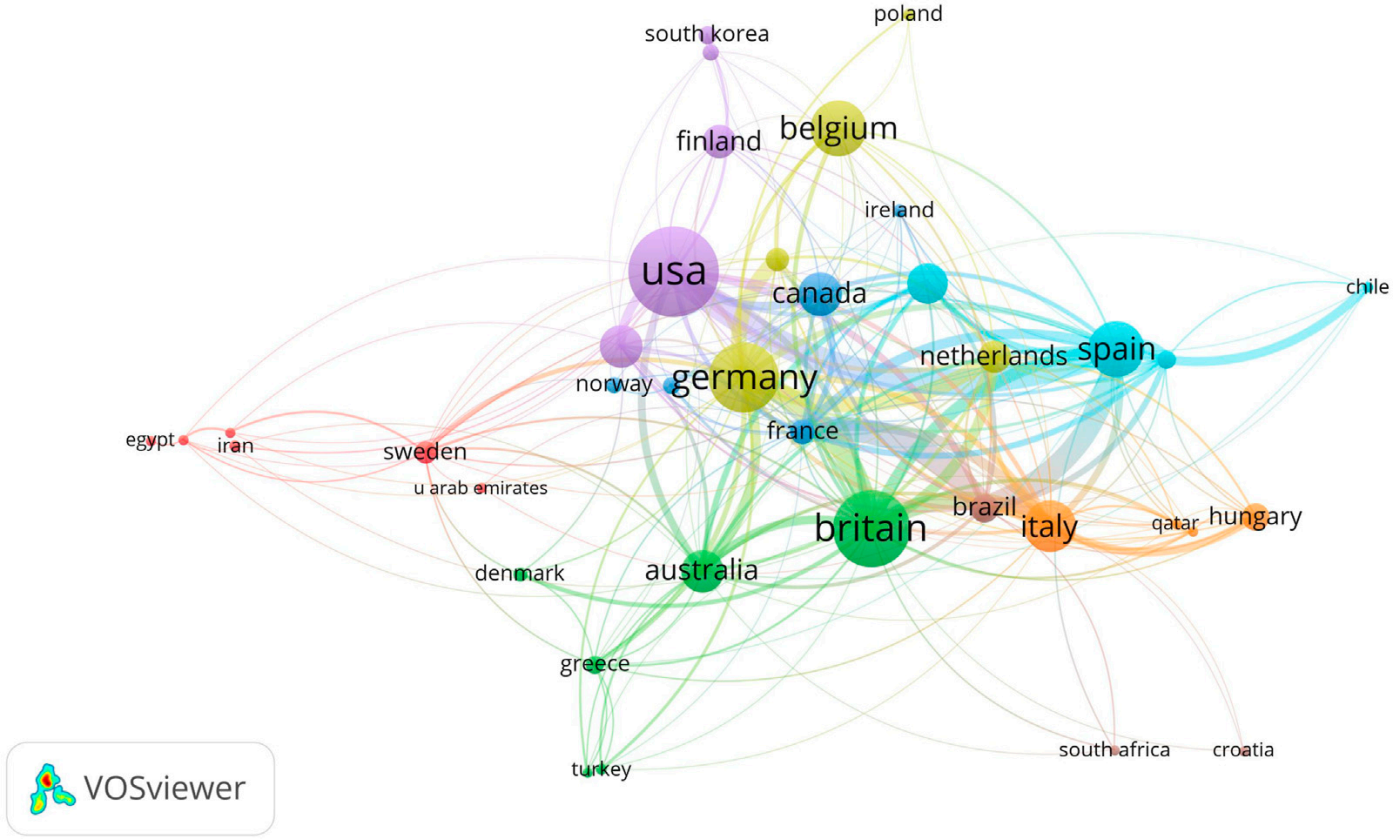
Cooperation network of the countries in the research of WBVT.

In addition to identifying the most influential countries in this field, a country cooperation analysis was also done. In the cooperative network of the countries, each element consists of a node and several links of varying thicknesses connected by the nodes (Zhao et al., 2019). In this study, the number of citations was chosen to represent the size of the nodes. The larger the node, the more influential the country is. The distance and thickness of the links between two countries describe the degree of cooperation. The minimum amount of publications of a country was set as 5. Of the 61 countries participating in the WBVT study, 38 countries met the threshold. Among the top 10 countries with the most significant publications, the United States and China (purple marked), Britain and Australia (green marked), and Canada and France (dark blue marked) were in the same cooperation network. The remaining four countries were in a cooperation network marked by different colors (Figure 2).

### Distribution and Co-Authorship of Organizations

Based on the web of science online bibliometric analysis, the 1,629 publications on WBVT originated from 1,770 organizations. In addition to the League of European Research Universities (LERU) and the Helmholtz association (their members have been calculated separately), the top 10 organizations (including 11 institutions) engaged in WBVT research have 397 records, accounting for 24% of the total number of publications (Table 3). Due to the cooperation among organizations, the top 10 organizations may publish less than 397 articles. University of Cologne (*n* = 53, 3.3%) in Germany and the Universidade do Estado do Rio de Janeiro (*n* = 52, 3.2%) in Brazil headed the list of organizations that indicated that they made a considerable effort in the field of WBVT. Except for the aforementioned two organizations, the top 5 organizations that published the most literature also included: Universite de Reims Champagne-Ardenne of France (*n* = 36, 2.2%), European University Miguel de Cervantes of Spain (*n* = 35, 2.1%), and The Hong Kong Polytechnic University of China (*n* = 35, 2.1%). Among the top 10 organizations, four were from Germany, and two were from Spain.

**TABLE 3:** Top 10 organizations that published the most publications on WBVT.

Rank	Organization	Country	Publication count	% of 1,629	Times cited
1	UNIVERSITY OF COLOGNE	Germany	53	3.254	963
2	UNIVERSIDADE DO ESTADO DO RIO DE ANEIRO	Brazil	52	3.192	272
3	UNIVERSITE DE REIMS CHAMPAGNE ARDENNE	France	36	2.210	1,338
4	MIGUEL DE CERVANTES EUROPEAN UNIVERSITY UEMC	Spain	35	2.149	828
4	HONG KONG POLYTECHNIC UNIVERSITY	China	35	2.149	703
6	MASSEY UNIVERSITY	New Zealand	33	2.026	1,195
6	UNIVERSITY OF SEVILLA	Spain	33	2.026	438
8	CHARITE UNIVERSITATSMEDIZIN BERLIN	Germany	32	1.964	593
8	GERMAN SPORT UNIVERSITY COLOGNE	Germany	32	1.964	537
10	KU LEUVEN	Belgium	28	1.719	3,027
10	GERMAN AEROSPACE CENTRE DLR	Germany	28	1.719	1,062

**FIGURE 3:**
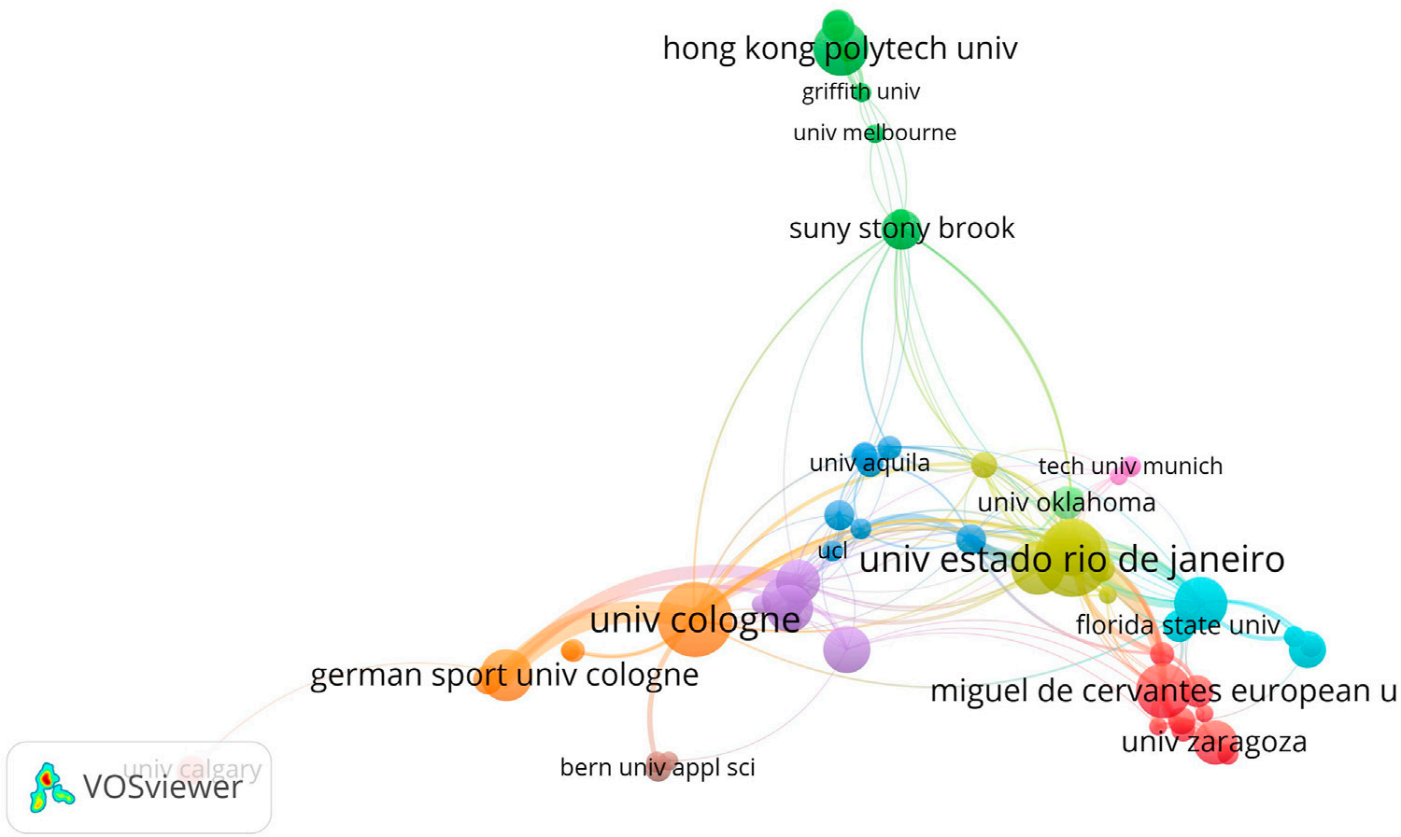
Cooperation network of the organizations in the research of WBVT.

VOSviewer constructs the knowledge domain map of WBVT research organizations to discover the current cooperation status of global research organizations. In this study, the node size indicates the number of literature files issued by the research organization. The link between the nodes (distance, thickness) represents the cooperation between research organizations. The minimum amount of literature was set as 8. 74 organizations met the threshold. We finally chose the most extensive set of related items consisting of 60 organizations after excluding some organizations without connection in the network. A pair of German organizations with the closest cooperation were the University of Cologne and German Sport University Cologne, with a link strength of 20. The link strengths of two pairs of organizations were followed closely, both of which were 12. They were the German Sport University Cologne and German Aerospace Center, Universidade do Estado do Rio de Janeiro in Brazil, and Istituto Auxologico Italiano (IRCCS) in Italy. Among the top 10 organizations that published the most, the University of Cologne and German Sport University Cologne were in the same cooperation network marked with orange, Universidade do Estado do Rio de Janeiro, Universite de Reims Champagne-Ardenne, and Massey University were in the same cooperation network marked with yellow, and Charite Universitatsmedizin Berlin, German Aerospace Center, and Katholieke Universiteit Leuven were in the same cooperation network marked with purple. Organizations worldwide were categorized into eleven color-coded categories (Figure 3).

### Distribution of Authors and Author Cooperation

There were more than 5,500 authors counted in this bibliometric study. Bernardo-Filho, Mario, from Universidade do Estado do Rio de Janeiro in Brazil, ranked first among the top 10 productive authors with 53 publications, followed by Marin, Pedro J (41 publications) from the European University Miguel de Cervantes in Spain and De Sa-caputo, Danubia Da Cunha (39 publications) from Universidade do Estado do Rio de Janeiro. At the same time, Rittweger, Joern was the most-times co-cited author with 1,098 co-citations, followed by Cardinale, Marco (1,053 co-citations) from the University of Aberdeen in Scotland and Bosco, Carmelo (873 co-citations) from the University of Rome Tor Vergata in Italy (Table 4).

**TABLE 4:** Top 10 productive authors and co-cited authors in the WBVT study.

Rank	Author	Publication count	Co-cited author	Co-citations
1	Bernardo M.	53	Rittweger J.	1,098
2	Marin P. J.	41	Cardinale M.	1,053
3	De Sa-caputo D. D.	39	Bosco C.	873
4	Rittweger J.	37	Cochrane D. J.	697
5	Felsenberg D.	34	Torvinen S.	651
6	Schoenau E.	34	Rubin C.	544
7	Taiar R.	31	Roelants M.	499
8	Cochrane D. J.	31	Delecluse C.	458
9	Sanudo B.	27	Marin P. J.	416
10	Lacerda ACR	27	Verschueren S.	384

**FIGURE 4:**
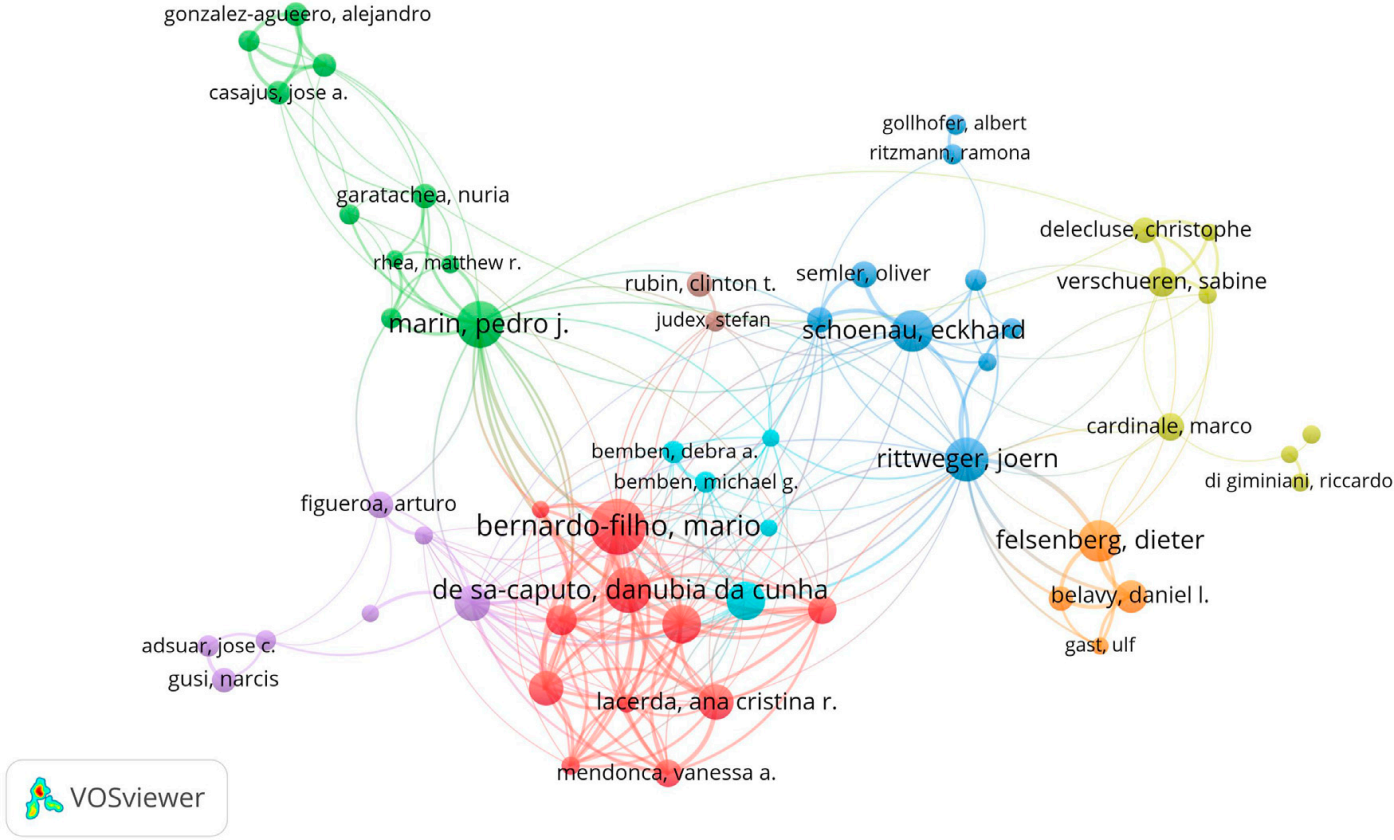
Co-authorship network of productive authors in the research of WBVT.

VOSviewer was used to generate a co-authors’ map displaying the distribution of the research groups in WBVT research. The minimum amount of literature of an author was set as 10. 69 authors met the threshold. Some of the 69 authors in the network were not connected. We chose the most extensive set of related items consisting of 56 authors. This knowledge domain map contained 56 nodes and 253 links (Figure 4). Each node represented an author, with the size of the node corresponding to the number of articles published by the author. The lines between the nodes indicated the cooperative relationship between authors. It can be seen from Figure 4 that researchers in the field of WBVT were composed of research groups of eight different colors. Moreover, the red research group was the core organization of the whole field, connecting the other research groups. Among the red’s core cluster, Bernardo-Filho, Mario was the author with the most literature and the most significant link strength with 240 articles.

### Distribution of Source Journals and Co-Cited Journals

According to the retrieved results, publications on WBVT research were published in 491 journals. Table 5 lists the top 10 journals with more than 20 publications on this topic. Most of the publishers of these journals are in the United States and Europe. The Journal of Strength and Conditioning Research (*n* = 110, 6.8%) topped the list of journals by a wide margin. Journals with more than 40 publications also included the Journal of Musculoskeletal and Neuronal Interactions (*n* = 51, 3.1%) and the European Journal of Applied Physiology (*n* = 47, 2.9%), ranking second and third.

**TABLE 5:** Top 10 productive journals in the WBVT study.

Rank	Journal	Publication count	% of 1,629	Citations	IF 2020	Country
1	Journal of strength and conditioning research	110	6.753	3,118	3.781	USA
2	Journal of musculoskeletal neuronal interactions	51	3.131	957	2.041	Greece
3	European journal of applied physiology	47	2.885	2,566	3.078	Germany
4	Plos one	36	2.210	571	3.240	USA
5	International journal of sports medicine	30	1.842	1,125	3.118	Germany
6	Journal of sports science and medicine	24	1.473	472	2.988	Turkey
7	Archives of physical medicine and rehabilitation	23	1.412	1,101	3.966	USA
8	Journal of sports medicine and physical fitness	22	1.351	313	1.637	Italy
9	Scandinavian journal of medicine science in sports	22	1.351	918	4.221	Denmark
10	Journal of electromyography and kinesiology	20	1.228	376	2.368	England

**FIGURE 5:**
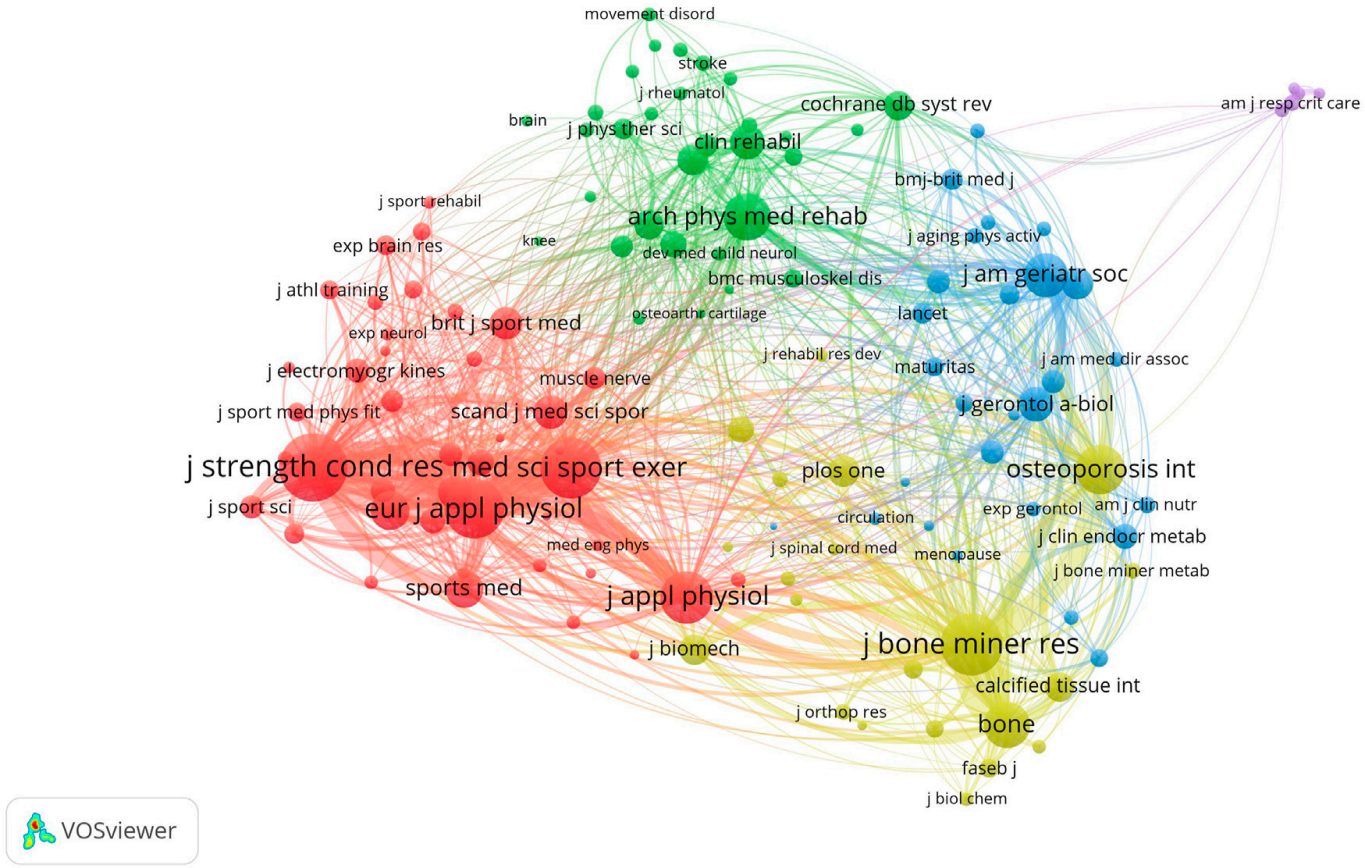
Co-citation network of productive journals in the research of WBVT.

VOSviewer generated a co-citation map of cited journals that published WBVT literature. The minimum amount of co-citations of a journal were set as 100. 126 journals met the threshold. This knowledge domain map contained 126 nodes and 7,226 links (Figure 5). Each node represented a journal, with the size of the node corresponding to the co-citations of the literature published by the journal. The lines between the nodes indicated the co-cited relationship between journals. Our statistical analysis revealed 5 co-citation clusters marked by different colors, as shown in Figure 5. In the top 10 productive journals, there were seven journals in the red cluster. Half of the top 10 journals with the highest total link strengths came from the red cluster. The most frequently co-cited journals were the Journal of Strength and Conditioning Research and Medicine and Science in Sports and Exercise, with a link strength of 12,104, followed by the Journal of Strength and Conditioning Research and European Journal of Applied Physiology, with a link strength of 11,874. Another pair of co-cited journals with a link strength of over 10,000 was the Journal of Bone and Mineral Research and Bone.

### Evolution and Main Path Analysis of Whole-Body Vibration Training

**FIGURE 6:**
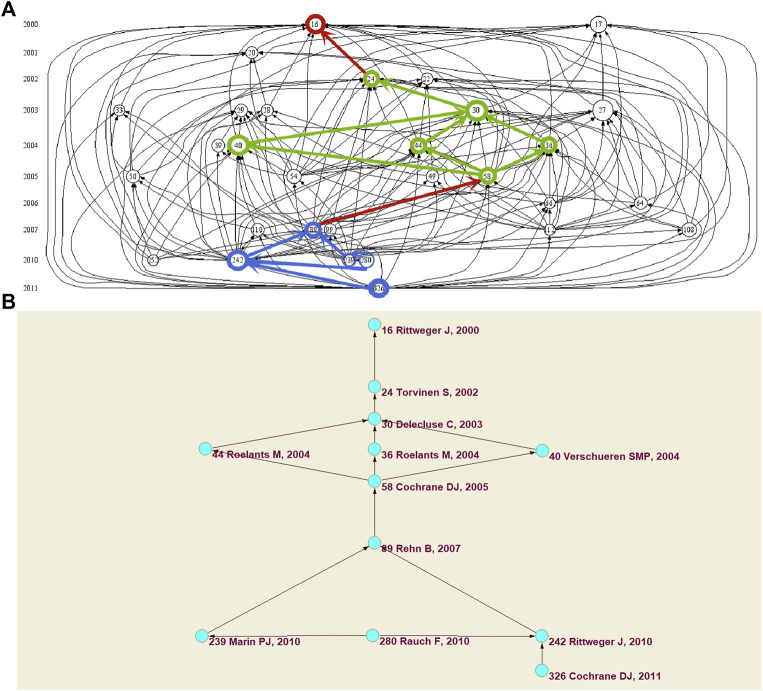
Evolution and the main path analysis of WBVT. **(A)** Citation chronology fusion map of the main path; **(B)** Main path of the citation chronology map.

Through the citation chronology map generated by HistCite Pro, we can observe the history of WBVT research, the citation relationship between literature, and each research stage in critical directions. The 30 pieces of literature with the highest citation frequency in the literature collection of WBVT were selected to draw the citation chronology map. Figure 6A shows the 30 pieces of literature in chronological order, which covered 2000–2011. Each node represented a piece of literature. The size of the node was directly proportional to the frequency of citation. The line with an arrow represented the reference relationship between the nodes. The literature pointed by the arrow was the cited literature. The chronological map of the citations showed the chronological order from top to bottom in a spatial order. In Figure 6A, the total number of links was 175, and the maximum value of the local citation score (LCS) was 363. The detailed information of the 30 pieces of literature is listed in Table 6.

**TABLE 6:** Node information of citation chronology map (LCS Count = 30).

Serial number	Author/Year/Journal/Volume/Page	LCS	GCS
16	Rittweger J, 2000, CLIN PHYSIOL, V20, P134	242	320
17	Bosco C, 2000, EUR J APPL PHYSIOL, V81, P449	236	337
20	Rittweger J, 2001, EUR J APPL PHYSIOL, V86, P169	126	162
22	Torvinen S, 2002, INT J SPORTS MED, V23, P374	121	165
24	Torvinen S, 2002, MED SCI SPORT EXER, V34, P1523	180	238
27	Cardinale M, 2003, EXERC SPORT SCI REV, V31, P3	354	503
28	Rittweger J, 2003, CLIN PHYSIOL FUNCT I, V23, P81	132	177
29	Torvinen S, 2003, J BONE MINER RES, V18, P876	141	212
30	Delecluse C, 2003, MED SCI SPORT EXER, V35, P1033	363	490
33	Russo CR, 2003, ARCH PHYS MED REHAB, V84, P1854	103	156
36	Roelants M, 2004, INT J SPORTS MED, V25, P1	150	186
39	Rubin C, 2004, J BONE MINER RES, V19, P343	150	405
40	Verschueren SMP, 2004, J BONE MINER RES, V19, P352	324	544
44	Roelants M, 2004, J AM GERIATR SOC, V52, P901	205	278
49	Luo J, 2005, SPORTS MED, V35, P23	142	212
50	Bruyere O, 2005, ARCH PHYS MED REHAB, V86, P303	153	226
54	Cardinale M, 2005, BRIT J SPORT MED, V39, P585	174	253
58	Cochrane DJ, 2005, BRIT J SPORT MED, V39, P860	168	251
64	Roelants M, 2006, J STRENGTH COND RES, V20, P124	143	199
66	Kvorning T, 2006, EUR J APPL PHYSIOL, V96, P615	115	143
89	Rehn B, 2007, SCAND J MED SCI SPOR, V17, P2	128	180
99	Bogaerts A, 2007, J GERONTOL A-BIOL, V62, P630	129	176
108	Abercromby AFJ, 2007, MED SCI SPORT EXER, V39, P1642	170	220
110	Abercromby AFJ, 2007, MED SCI SPORT EXER, V39, P1794	136	194
112	Hazell TJ, 2007, APPL PHYSIOL NUTR ME, V32, P1156	104	136
239	Marin PJ, 2010, J STRENGTH COND RES, V24, P548	111	130
242	Rittweger J, 2010, EUR J APPL PHYSIOL, V108, P877	301	459
252	Machado A, 2010, SCAND J MED SCI SPOR, V20, P200	111	151
280	Rauch F, 2010, J MUSCULOSKEL NEURON, V10, P193	172	216
326	Cochrane DJ, 2011, INT J SPORTS MED, V32, P75	111	163

The main path of the citation chronology map in the WBVT research field from 2000 to 2011 was obtained from Pajek, as shown in Figure 6B. Consistent with the citation chronology map, the node represented the literature, and the literature’s serial number, author, and publication year were marked. The direction of the arrow indicated the citation relationship between the latter and the former. The literature from 2000 to 2011 was linked through a series of nodes. The whole main path expressed the development key nodes, research directions, and development trends of WBVT research. Figure 6A intuitively shows the reference relationship between the main path and the other literature in this field.

### Research Trends and Hot Spot Analyses of Whole-Body Vibration Training

**FIGURE 7:**
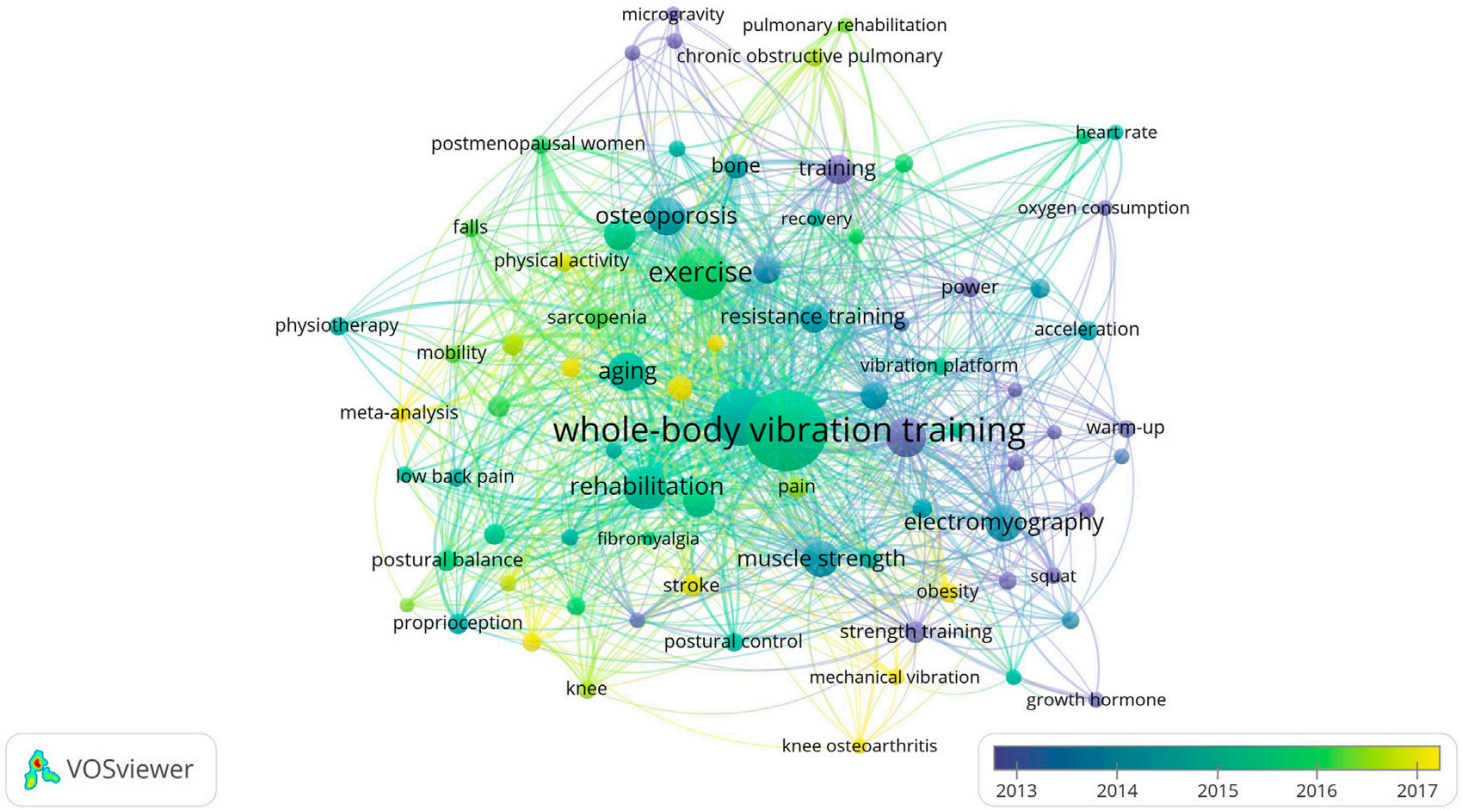
Co-occurrence clustering network of keyword–time dual-map in the research of WBVT.

We used the author keyword co-occurrence network to construct the knowledge map of WBVT research to predict the development trend of this field. The co-occurrence clustering map of author keywords varying over time by VOSviewer with a frequency greater than 12 is shown in Figure 7. After excluding duplicates, 80 high-frequency keywords were displayed in different color gradients according to the year of occurrence. The average publication age of most keywords was around 2015. The lighter the color, the more recently the word appeared. In Table 7, the keywords appearing after 2015 focused on the researchers’ attention to WBVT in recent years. It may indicate the research trend in this field to a certain extent.

**TABLE 7:** Keywords appearing after 2015 in the WBVT study.

No.	Author keyword	Average year of publication	Frequency	Total link strength
1	Mechanical vibration	2019.29	15	18
2	Knee osteoarthritis	2018.69	13	20
3	Physical exercise	2018.46	15	25
4	Exercise therapy	2017.95	20	40
5	Meta-analysis	2017.75	16	45
6	Vibration therapy	2017.25	21	37
7	Quality of life	2017.15	35	83
8	Obesity	2017.09	22	44
9	Stroke	2016.88	26	54
10	Physical activity	2016.78	21	38
11	Randomized controlled trial	2016.64	15	42
12	Systematic review	2016.56	27	69
13	Chronic obstructive pulmonary disease	2016.50	20	46
14	Knee	2016.44	18	44
15	Pain	2016.35	23	50
16	Physical therapy	2016.25	12	17
17	Pulmonary rehabilitation	2016.23	13	31
18	Mobility	2016.18	17	49
19	Falls	2016.06	18	53
20	Sarcopenia	2016.03	33	80
21	Cerebral palsy	2015.98	27	66
22	Body composition	2015.86	14	36
23	Postmenopausal women	2015.85	20	63
24	Blood pressure	2015.85	12	27
25	Exercise	2015.81	182	383
26	Osteoarthritis	2015.74	20	47
27	Spinal cord injury	2015.72	19	43
28	Postural balance	2015.63	27	62
29	Fibromyalgia	2015.58	12	25
30	Bone mineral density	2015.43	60	161
31	Balance	2015.43	60	141
32	Whole-body vibration training	2015.37	460	680
33	Range of motion	2015.31	13	15
34	Vibration platform	2015.25	16	28
35	Gait	2015.24	25	64
36	Maximal voluntary contraction	2015.08	13	19
37	Biomechanics	2015.07	14	20
38	Postural control	2015.00	19	39

**FIGURE 8:**
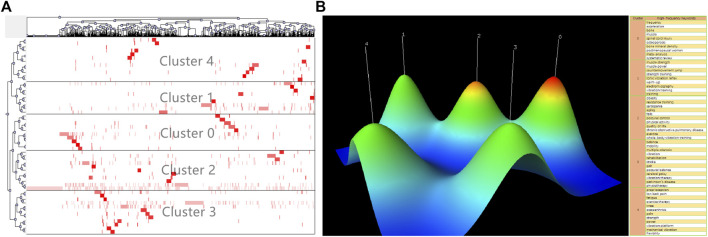
Biclustering analysis of the high-frequency word–paper matrix on WBVT. **(A)** Tree visualization; **(B)** Mountain visualization.

We also used the clustering analysis to grasp the research hot spots of WBVT. A total of 2,509 keywords were extracted from the literature by BICOMB. At the same time, keywords with the same meaning were combined to ensure the accuracy and reliability of the data. BICOMB extracted 54 high-frequency keywords with a frequency greater than or equal to 16, and the word–paper matrix of high-frequency keywords was extracted in the matrix analysis. The word–paper matrix constructed by BICOMB was imported into gCLUTO for the biclustering analysis. In order to determine the best clustering results, biclustering was repeated several times by adjusting the number of clusters (Lu et al., 2018). The clustering results with relatively high average Isim (Internal Similarities) and relatively low average Esim (External Similarities) were selected, and the final clustering number was determined to be 5. Figure 8 is a biclustering visualization formed by high-frequency keywords for rows and co-occurrence literature for columns. There were five relatively independent and distributed mountains, representing five hot spots in WBVT research. The peaks of clusters 0 and 2 were red, indicating highly consistent research topics.

## Discussion

The present study is the first bibliometric analysis for WBVT. We observed a rapid increase in the number of publications and citations from 2000 to 2022, which indicates that WBVT has received increasing attention as a method of exercise rehabilitation.

Through the bibliometric analysis of the countries where the literature is located, we can understand the attention and contribution of different countries to the relevant research fields. In addition to identifying the most influential countries in this field, a country cooperation analysis reflects the degree of co-authorship between regions (Zhao et al., 2019). Spain had the highest total link strength in the present study, followed by Germany, Britain, and the United States. The literature output of these four countries ranked among the top 5 in the world. China ranked third in output, but the total link strength was low, indicating that there was relatively little international cooperation. The co-authorship between the United States and Spain was the closest. Brazil also had close cooperation with France and Spain, closely following the United States and Spain. Germany and Britain were slightly behind them, ranking fourth in link strength. The total link strength of France was 116, but the frequency of citations was only 881. Although Belgium’s total link strength was only 35, the times cited had reached 3,814. There seems to be no absolute relationship between international cooperation and influence.

Analyzing the distribution and co-authorship of research institutions can provide a reference basis for academic exchanges and selecting, training, and promoting talents. To further discover the current cooperation status of global research organizations, the knowledge domain map of WBVT research organizations was constructed. Among the top 10 organizations, Charite Universitatsmedizin Berlin, German Sport University Cologne, and German Aerospace Center were all from Germany. So, the aforementioned research institutions in Germany may be the better choice for academic exchanges and talent training. This study showed that the frequency of cooperation between WBVT research organizations in the world was low. It is worth noting that the cooperation of domestic organizations still dominated the form of cooperation. The closest cooperation was between the University of Cologne and German Sport University Cologne. There were only a few relatively stable cooperation groups with countries as units in the world. Therefore, researchers worldwide should increase communication and promote global cooperation for a more rapid development of WBVT research.

The research on the distribution of authors can grasp the breadth and depth of research in this field. The distribution of authors is the epitome of scientific research. The prosperity and development of WBVT research are inseparable from a group of core researchers with a solid theoretical foundation and rich achievements. We generated a co-authors’ map displaying the distribution of the research groups in WBVT research. Bernardo-Filho, Mario, who had the most literature outputs and a total link strength of 240 could play a critical role in the international cooperation and discipline development of the WBVT field. However, we found that among the top 10 authors cited frequently, no authors were in the red cluster of high- productivity authors. The most frequently cited author was Verschueren, Sabine (2,883 citations) from Katholieke Universiteit Leuven of Belgium in the yellow cluster, followed by Rittweger, Joern (2,575 citations) from the Free University of Berlin in the dark blue cluster. Although the number of some cluster members was small and the literature output was not ranked in the top 10, the citation frequency of all co-authors in the cluster was relatively high. For example, there were only two co-authors (Rubin, Clinton T, and Judex, Stefan from SUNY Stony Brook University in the United States) in the brown cluster, all cited in the top 10.

Analyzing the sources of publications can help the researchers in the field of WBVT choose a better submission scheme and help researchers select and read appropriate references to improve the pertinence and effectiveness of WBVT research. The vital knowledge source and core journals in an academic field can be mastered by analyzing the co-citation of journals in that field. We generated a co-citation map of cited journals that published the WBVT literature. The most frequently co-cited journals were all from the red cluster. Another pair of frequently co-cited journals was the Journal of Bone and Mineral Research and Bone from the yellow cluster. They were not in the top 10, but the co-cited analysis showed that the two journals had a specific influence in this field. Researchers in WBVT may obtain valuable information about WBVT from these journals. These journals also became the primary choice for the submission on WBVT.

The main path described the knowledge bases of WBVT research. In the present study, the main path contained 12 key nodes, starting from Rittweger’s study on the acute physiological effects of WBVT in 2000 (Rittweger et al., 2000). WBVT research was basically maintained on one main path, which was divided into two stages. The first stage ran from 2002 to 2005 and contained six important node literature pieces. This stage began with Torvinen’s research on the effects of whole-body vibrations on muscle performance and body balance in 2002 (Torvinen et al., 2002). In 2003, after Delecluse proposed that WBVT could increase female knee-extensor strength (Delecluse et al., 2003), this route was divided into three branches in 2004. These three branches were to study the effects of WBVT on women’s athletic ability. Two of these studies came from Roelants, one on muscle strength (Roelants et al., 2004a) and the other on the strength of knee-extension and the speed of movement (Roelants et al., 2004b). The third was Verschueren’s research on hip density and postural control in addition to muscle strength (Verschueren et al., 2004). The three branches converged again into the main path in 2005. That year, Cochrane found that acute WBVT improved flexibility performance and vertical jump in female hockey athletes (Cochrane and Stannard, 2005). From 2007 to 2011, the second stage contained five important node literature pieces, which began with the effect of WBVT on leg muscle performance proposed by Rehn (Rehn et al., 2007). Then, this stage was divided into two branches in 2010. Rittweger reviewed the research process of WBVT from theory to practical application and discussed its possible potential applications in sports and medicine (Rittweger, 2010). The other branch was Marin’s research on the effect of vibration training on muscle strength (Marín and Rhea, 2010). Based on these two studies, the International Society of Musculoskeletal and Neuronal Interactions (ISMNI) invited experts in this field to provide suggestions on how to describe interventions in the reports about WBV treatment studies (Rauch et al., 2010). In 2011, Cochrane continued to support Rittweger’s research on the potential benefits of WBVT and further proposed that the study should focus on the optimal dose relationship of amplitude, frequency, and duration in different populations (Cochrane, 2011). A map of the critical literature nodes of the main path obtained by Pajek to the citation chronology map obtained by HistCite Pro could help to obtain the citation chronology fusion map of the main path (Dan et al., 2021). We next obtained the citation chronology fusion map of the main path. We found that the literature on the main path may not be cited most frequently. However, the literature put forward critical theories or innovative concepts in WBVT research (Dong and Guo, 2010).

Keywords can express the literature theme, and the clustering analysis of co-occurrence keywords can reveal the hot spots in this research field (Zhao et al., 2019). Through the age of keywords, we can predict the research trend of a discipline in recent years. The author keywords are the most primitive natural language proposed by the authors of the publications without standardization, which may best represent the emerging theme of a subject. In this study, we found that the early studies were focused on postural control, balance, bone mineral density, and body composition, which indicated that many studies aimed to find evidence to support the use of WBVT to improve athletic performance in the past (Beck, 2015; Cloak et al., 2016; Gómez-Bruton et al., 2017). The keywords used in more recent articles were obesity, stroke, pulmonary rehabilitation, sarcopenia, and quality of life, which revealed that WBVT was studied for the application in the exercise rehabilitation of several chronic diseases in recent years (Pleguezuelos et al., 2013; Silva et al., 2014; Alvarez-Alvarado et al., 2017; Wu et al., 2020). Studies related to the previous keyword “osteoarthritis” and the recent keyword “knee osteoarthritis” showed that the application of WBVT in osteoarthritis has always been the focus. Our data showed that the research trends in WBVT studies identified by analyzing the keywords in terms of time were exercise rehabilitation of chronic diseases, especially knee osteoarthritis in the future. A biclustering analysis is to classify individuals or objects according to their similarities and help us grasp research hot spots and development trends. According to the clustering results, the present study obtained five hot spots in WBVT research. The main keywords involved in the five hot spots were aging, exercise, sarcopenia, osteoporosis, stroke, low back pain, osteoarthritis, chronic obstructive pulmonary disease, quality of life, etc. It is confirmed that the exercise rehabilitation of several aging chronic diseases was the hot spot of WBVT.

The present study has some limitations. First, we mainly conducted the searches in WoSCC. Combining the results with those from other databases, such as PubMed and Scopus, would be better. Second, due to the collection characteristics of WoSCC, English literature accounts for 97% of the final collected literature. To some extent, this ignores the literature research published in other languages. Third, the number of clusters in the network analysis can be affected by the author’s subjective viewpoint. Fourth, we might have underestimated the contribution of recently published studies because of their low citation frequency. Fifth, due to the authors’ duplicate names and authors’ multiple units, there might be statistical errors in analyzing the authors and organizations.

## Conclusion

In conclusion, the present study provided valuable data for potential collaborations among researchers and institutions and identified hot spots and trends in the research on WBVT. Our data will contribute to identifying the core target diseases of WBVT and providing vital insights for researchers in this field.

## Data Availability

The original contributions presented in the study are included in the article/Supplementary Material; further inquiries can be directed to the corresponding author.
